# A Role for the Mitochondrial Protein Mrpl44 in Maintaining OXPHOS Capacity

**DOI:** 10.1371/journal.pone.0134326

**Published:** 2015-07-29

**Authors:** Janet H. C. Yeo, Jarrod P. J. Skinner, Matthew J. Bird, Luke E. Formosa, Jian-Guo Zhang, Ruth M. Kluck, Gabrielle T. Belz, Mark M. W. Chong

**Affiliations:** 1 Walter and Eliza Hall Institute of Medical Research, Parkville, VIC, Australia; 2 Department of Medical Biology, University of Melbourne, Parkville, VIC, Australia; 3 St. Vincent’s Institute of Medical Research, Fitzroy, VIC, Australia; 4 Murdoch Childrens Research Institute, Parkville, VIC, Australia; 5 Department of Paediatrics, University of Melbourne, Parkville VIC, Australia; 6 Department of Biochemistry, La Trobe University, Bundoora, VIC, Australia; 7 Department of Medicine (St Vincent’s), University of Melbourne, Parkville VIC, Australia; Newcastle University, UNITED KINGDOM

## Abstract

We identified Mrpl44 in a search for mammalian proteins that contain RNase III domains. This protein was previously found in association with the mitochondrial ribosome of bovine liver extracts. However, the precise Mrpl44 localization had been unclear. Here, we show by immunofluorescence microscopy and subcellular fractionation that Mrpl44 is localized to the matrix of the mitochondria. We found that it can form multimers, and confirm that it is part of the large subunit of the mitochondrial ribosome. By manipulating its expression, we show that Mrpl44 may be important for regulating the expression of mtDNA-encoded genes. This was at the level of RNA expression and protein translation. This ultimately impacted ATP synthesis capability and respiratory capacity of cells. These findings indicate that Mrpl44 plays an important role in the regulation of the mitochondrial OXPHOS capacity.

## Introduction

Almost all eukaryotic cells contain mitochondria, double membrane-enclosed structures that are crucial for the production of energy, in the form of ATP, by oxidative phosphorylation (OXPHOS). The OXPHOS system, encoded by genes within both the mitochondrial and nuclear genomes (mtDNA and nDNA), is comprised of 88 protein subunits that assemble into five multi-subunit complexes (Complex I—V), to form the electron transport chain at the inner membrane of the mitochondrion [1]. The system functions to link the oxidation of reduced coenzymes (NADH at Complex I, and FADH_2_ at Complex II), with proton pumping (at Complex I, III and IV) to generate the mitochondrial membrane potential (ΔΨ_m_). Complex V then utilizes the ΔΨ_m_ by mechanically coupling the flow of protons through the complex down their electrochemical gradient, driving ATP synthesis from ADP and inorganic phosphate.

The mitochondrion is thought to have evolved from free-living bacteria engulfed by an ancestral archaeal cell. The presence of the mtDNA supports this theory [2]. The mammalian mtDNA is a circular, double-stranded genome that encodes for 13 open reading frames (ORFs), 22 transfer RNAs (tRNAs) and 2 ribosomal RNAs (rRNAs) [3]. The 13 ORFs encode for proteins that are integral subunits of the OXPHOS system [4]. Defects in mtDNA transcription and translation are associated with numerous classical mitochondrial diseases including: chronic progressive external ophthalmoplegia; mitochondrial encephalopathy, lactic acidosis and stroke-like episodes; Leigh syndrome; myoclonic epilepsy with ragged red fibres; mitochondrial neuro-gastro-intestinal encephalomyopathy; and Pearson syndrome [5].

Apart from the mtDNA-encoded proteins, the majority of the proteins found in the mitochondria are nuclear-encoded. This includes proteins crucial for mtDNA replication, transcription and translation as well as proteins associated with the mitochondrial ribosome (mitoribosome), including proteins known as mitochondrial ribosomal proteins [6]. The mammalian mitoribosome is made up of a large subunit (39S), comprising the mtDNA-encoded 16S rRNA and 52 nuclear-encoded proteins (Mrpl), including a mitochondrial valine tRNA (tRNA^Val^), a crucial structural component, and a small subunit (28S), containing the mtDNA-encoded 12S rRNA and 31 nuclear-encoded proteins (Mrps) [7] [8–13].

Until recently, Drosha and Dicer were the only two known RNase III proteins in animals. These play important roles in the biogenesis of small non-coding RNAs, particularly microRNAs [14]. RNase III proteins are divalent metal ion-dependent phosphodiesterases that specifically bind to and cleave double stranded (ds) RNA. The RNase III domain dimerizes to form a structure that binds dsRNA and cleaves the phosphodiesters on each strand, resulting in products with characteristic 2 nucleotide (nt) 3’- overhangs [15]. This step, essentially the processing of dsRNA by RNase III enzymes, is crucial for the maturation and decay of both coding and non-coding RNAs [16,17].

Here, we investigate the function of a poorly characterized protein previously identified as a component of the mitoribosome. We show that Mrpl44 has a role in the regulation of mitochondrial gene expression and ultimately in the regulation of OXPHOS.

## Materials and methods

### Cell culture

Murine fibroblast (NIH3T3), mammary epithelial (CommaD P24), neuronal (N2A) and human embryonic kidney (HEK293T) cell lines were grown in Dulbecco’s Modified Eagle’s Medium (DMEM) supplemented with 10% fetal calf serum, penicillin, streptomycin and sodium pyruvate.

HEK293T cells were transfected by calcium phosphate method for co-immunoprecipitation (co-IP) experiments, and for producing retroviral/lentiviral supernatants. MSCV-based retroviruses encoding FLAG or GFP-tagged constructs or lentiviruses encoding shRNAs [18] were then transduced into the various cell lines. Short hairpin (sh) RNAs targeting the sequences at the 3’ UTR (5’TCTCTTACACACTGGTTTATTACT-3’) and the open reading frame (5’-GGAAAGAGCTCTTTGAGATGT-3’) were employed to knockdown Mrpl44. The murine sequence was employed for all constructs.

### Immunofluorescence microscopy

Cells expressing free GFP or Mrpl44GFP fusion were grown on gelatin-coated coverslips. MitoTrackerRed was added to the cultures 30min prior to harvesting to stain the mitochondria. The cells were then washed with PBS and fixed with paraformaldehyde, then stained with an αGFP antibody (#2956, Cell Signaling Technologies). DAPI was used to visualize the nucleus. Images were captured on a DeltaVision Elite widefield fluorescence microscope.

### Subcellular fractionation

Cells were fractionated into nuclei, cytosol (± heavy membranes), mitochondria and mitochondrial components. Nuclear versus cytoplasmic fractions were prepared as previously described [19]. The mitochondrial fraction was extracted with digitonin, as previously described [20].

### Co-immunoprecipitations (Co-IP)

Transiently transfected HEK293T cells and stably transduced NIH3T3 cells were used for analyses. Heavy membrane fractions were obtained by digitonin permeabilization and lyzed in KALB lysis buffer (20mM Tris-HCl pH 8.0, 100mM KCl, 0.2 mM EDTA). Supernatants were either co-IPed with M2 αFLAG agarose beads (Sigma), with a mouse αGFP antibody (1A12-6-18, produced in house), or with a rabbit αMrpl44 antibody (Proteintech) followed by Protein G sepharose beads (GE Healthcare). Bound proteins were eluted and analyzed by SDS-PAGE followed by Western blotting. Details of the antibodies used are provided in S1 Table. For the analysis of protein-RNA interactions, RNA was extracted with TriSure (Bioline) following the washing of the beads.

### RNA analyses

RNA was reverse transcribed with MLV-RT (New England Biolabs) and quantitated by quantitative PCR on a ViiA7 Real-Time PCR System (Life Technologies) with SYBR Green detection (Promega). The primer pairs employed are listed in S2 Table.

### Gel Filtration Chromatography

Whole cell extracts were fractionated using a Waters HPLC system on a pre-packed Superose 6 gel-filtration column (300mm x 10mm internal diameter, GE Healthcare) equilibrated in 0.5% Triton X-100 in 20mM Tris-HCl, pH7.5, 150mM NaCl containing protease inhibitors. The column was calibrated with standards (Bio-Rad), ranging from 670kDa to 1.35kDa. 0.6mL fractions were collected at a flow rate of 0.4mL/min.

### Mitochondrial translation assay

The analysis of mitochondrial gene translation was performed as previously described [21]. In brief, NIH3T3 cells were plated such that they reached a confluency of 75 to 90% on the day of the experiment. The cells were washed in PBS and pre-incubated with methionine/cysteine-deficient DMEM, supplemented with 10% dialyzed fetal bovine serum. The addition of emetine was used to inhibit cytoplasmic translation. EasyTag Expre^35^S^35^S Protein Labeling Mix [^35^S] (>1,000Ci/mmol) (Perkin Elmer) was then added to the wells and incubated for 120mins, with samples collected at 0, 30, 60 and 120mins. Protein was quantified by Micro-BCA (Thermo Scientific), and resolved on a 10–16% gradient gel (Biorad). The proteins were then electrotransferred to PVDF membrane for autoradiography. Quantification of radioactive counts was performed with ImageQuant TL 8.1 (GE Healthcare).

### Measurement of ATP synthesis and bioenergetics

ATP synthesis rates were measured in technical duplicates as previously described [22]. Briefly, the cells were diluted 10 fold in ATP synthesis buffer containing 50μg/ml digitonin and substrates with/without addition of the following substrates and inhibitors: succinate (10mM), malonate (1mM), glutamate (10mM), malate (10mM), rotenone (2.5μM). After 20min, the reactions were stopped with perchloric acid on ice, and neutralized with potassium hydroxide and MOPS. ATP concentration in the samples was determined by ATP Bioluminescence Assay (Roche).

The oxygen consumption (OCR) and extracellular acidification rates (ECAR) were measured using the Seahorse XF-24 Extracellular Flux Analyzer as previously described [23]. The various parameters were determined following the inhibition or uncoupling of the OXPHOS system components: rotenone (Complex I inhibitor), oligomycin (Complex V inhibitor), FCCP (ΔΨ_m_ uncoupler), and Antimycin A (Complex III inhibitor).

## Results

### Mrpl44 is a putative RNase III enzyme

In an attempt to identify additional RNase III enzymes in animals, we performed a search for proteins with sequence homology to the RNase III domain and double-stranded RNA-binding motif (dsRBM) of murine Drosha (S1 Fig). This analysis yielded a small protein, Mrpl44, which has a predicted molecular weight of 38kDa and is structurally similar to bacterial RNase III (S1 Fig).

Mrpl44 is a protein that was previously identified as part of the large subunit of the mitoribosome in bovine mitochondrial extracts [8]. It is conserved across a wide range of species and homologues can be found in yeast, nematodes, flies and humans, but not in bacteria [8]. However, the RNase III domain itself does not appear to be as well conserved as the RNase III domains of Drosha and Dicer [8].

Interestingly, *MRPL44* was recently found to be the gene mutated in a sib-pair with mitochondrial infantile cardiomyopathy [24]. This resulted in reduced MRPL44 expression levels in heart and skeletal muscles, with reduced MRPL44 expression appearing to affect assembly of the large ribosomal subunit and stability of the 16S RNA [24].

### Mrpl44 is localized to the mitochondria

The conservation of MRPL44 across a wide range of organisms suggests that it plays an important function in cells. While MRPL44 was originally found to be associated with the mitochondria, it has also been captured with a wide variety of cytoplasmic and nuclear baits suggesting that it may not be specifically localized to the mitochondria [25].

**Fig 1:**
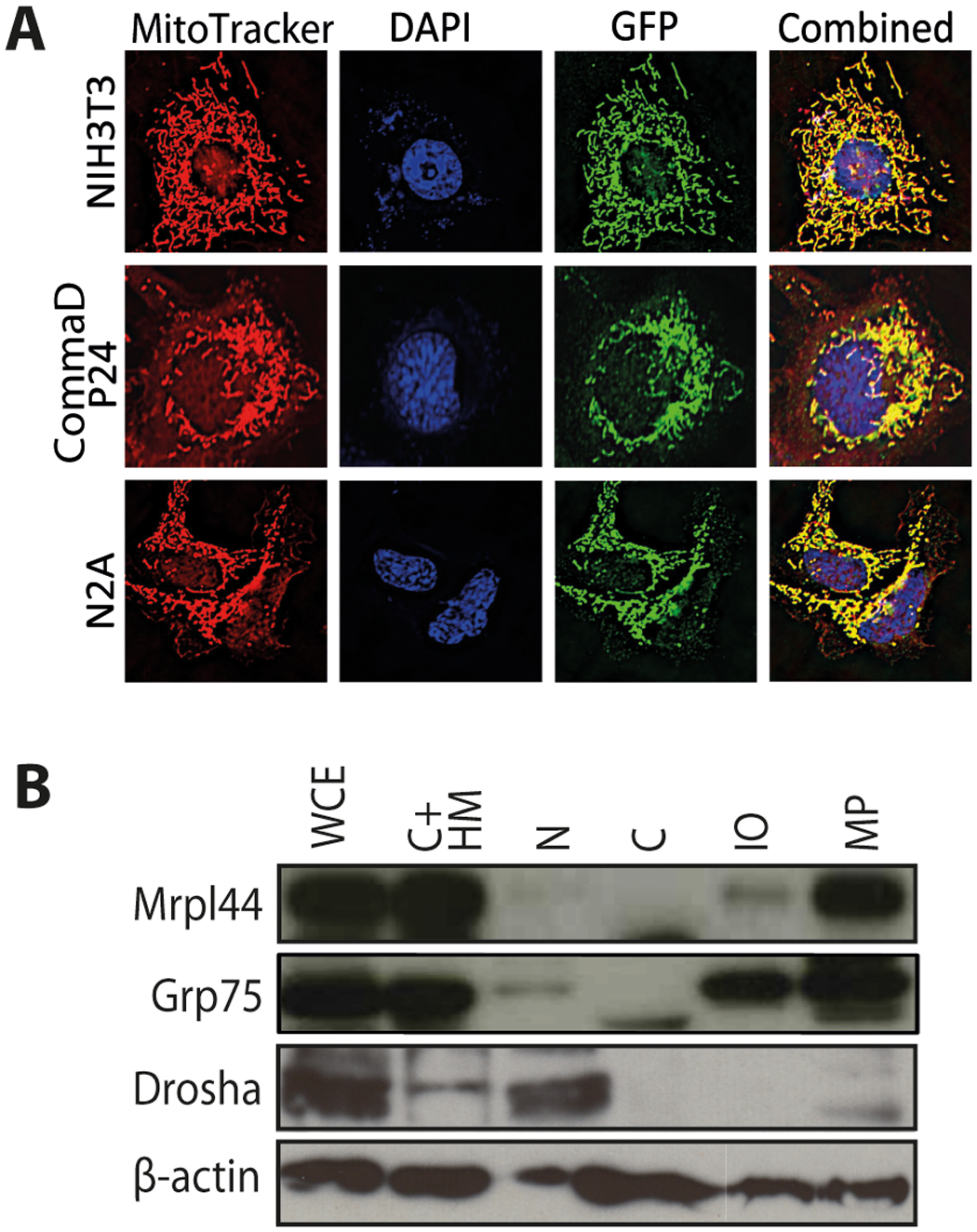
Mrpl44 is predominantly localized to the mitochondria. (A) Shown are NIH3T3 fibroblasts, CommaD P24 mammary epithelial cells and N2A neuronal cells expressing an Mrpl44GFP fusion. The cells were co-stained with MitoTrackerRed along with DAPI and analyzed by wide-field microscopy (400X magnification). (B) NIH3T3 cells were sub-fractionated into the following fractions: cytosol and heavy membranes (C+HM), nuclear (N), cytosol (C), mitochondrial outer membrane and intermembrane space (IO) and mitoplast (MP). Whole cell extract (WCE) is also shown. Fractions were blotted with antibodies against Mrpl44 as well as Grp75, a known mitochondrial protein, Drosha, a nuclear protein, and β-actin, a cytoskeletal protein.

To better define the cellular localization of Mrpl44, we generated an Mrpl44GFP fusion construct and transduced it into the cell lines NIH3T3, CommaD P24 and N2A cells. Mrpl44GFP co-localized with MitoTrackerRED in all cell lines examined (Fig 1A). We also examined for co-localization with PD1 in the endoplasmic reticulum and phalloidin, which binds filamentous actin, but found no co-localization (data not shown). This indicates that Mrpl44GFP is specifically localized to the mitochondria. In contrast, untagged GFP showed no specific localization (S2 Fig)

We further confirmed that endogenous Mrpl44 is localized to the mitochondria. NIH3T3 fibroblasts were sub-fractionated into nuclear, cytosol plus heavy membrane, cytosol only, mitochondrial outer membrane and intermembrane space (IO) and the mitoplast (MP, mitochondrial inner membrane plus matrix). The presence of endogenous Mrpl44 was analyzed by Western blotting. We found that Mrpl44 was present in all fractions containing mitochondria, but was predominantly localized to the MP fraction (Fig 1B).

### Mrpl44 forms multimers as part of the large subunit of the mitochondrial ribosome

**Fig 2:**
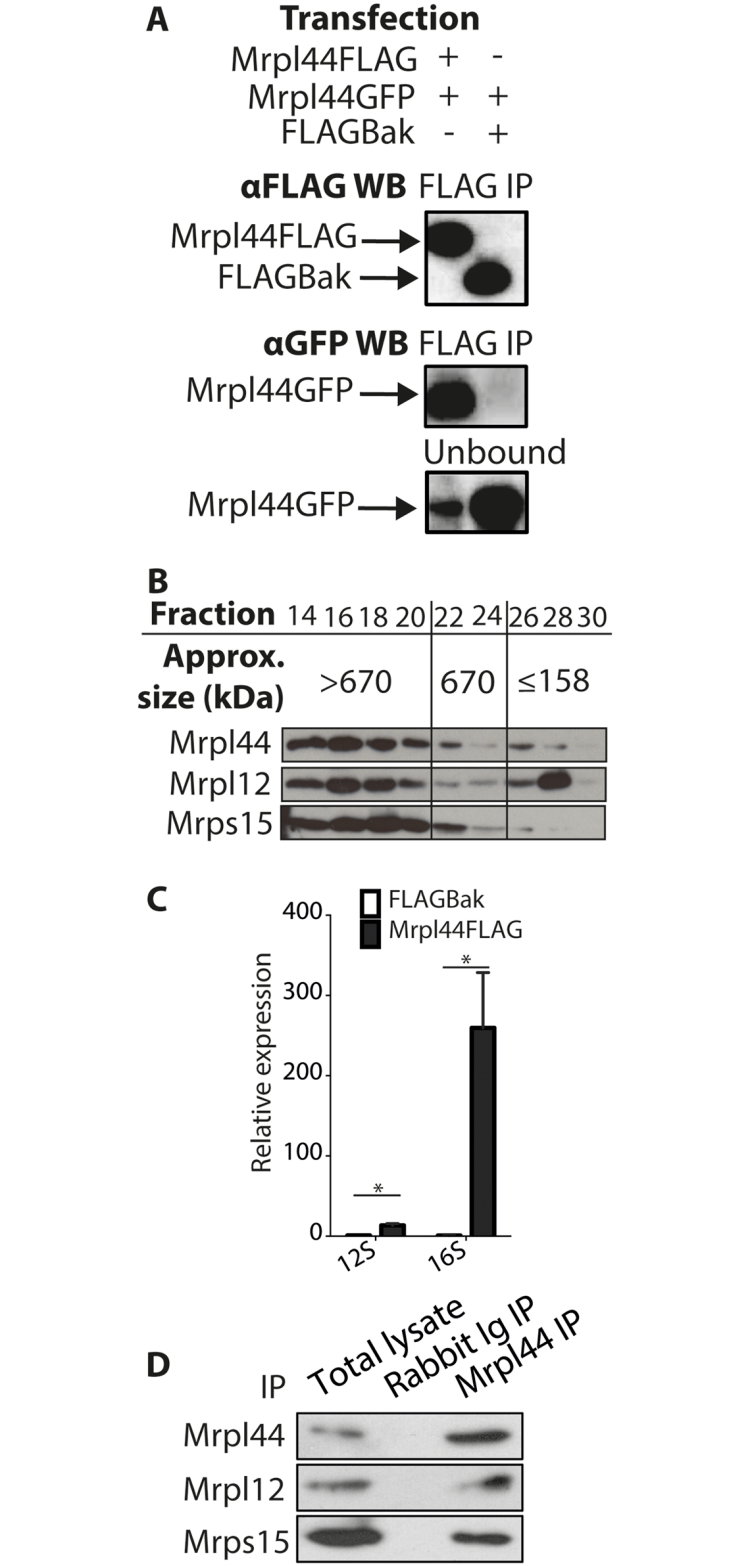
Mrpl44 forms multimers as part of the large subunit of the mitoribosome. (A) HEK293T cells were co-transfected with Mrpl44FLAG and Mrpl44GFP; or FLAGBak and Mrpl44GFP, as a negative control. Lysates were immunoprecipitated with αFLAG, then blotted with an αGFP antibody to determine co-IP. Results are representative of three independent experiments. (B) NIH3T3 whole cell extracts were fractionated by HPLC on a Superose 6 column. The fractions were collected and alternate fractions from 14 to 30 were run on an SDS-PAGE gel, then blotted with antibodies against Mrpl44 as well as Mrpl12 and Mrps15, known components of the mitoribosome. (C) Mitochondrial fractions were obtained from Mrpl44FLAG NIH3T3 cells, and FLAGBak NIH3T3 cells, as a negative control, and immunoprecipitated with αFLAG agarose beads. RNA was then extracted and analysed for pulldown of the mitochondrial rRNA subunits by quantitative RT-PCR. The mean +/- SEM of three independent experiments is shown. Statistical analysis performed using multiple t-test with Holm-Sidak correction for multiple comparisons (*p<0.05). (D) NIH3T3 whole cell extracts were immunoprecipitated with αrabbit Ig antibody or αMrpl44 antibody bound to Protein G sepharose beads then blotted for Mrpl44, Mrpl12 and Mrps15.

RNase III proteins typically form dimers that bind to and cleave dsRNA [15]. To determine whether Mrpl44 might form dimers or multimers, FLAG-tagged Mrpl44 (Mrpl44FLAG) was co-transfected with Mrpl44GFP into HEK293T cells. As a negative control, Mrpl44GFP was co-transfected with FLAGBak, another mitochondria-localized protein. Lysates were immunoprecipitated with αFLAG beads to capture Mrpl44FLAG or FLAGBak, then Western blotted to determine if Mrpl44GFP was co-immunoprecipitated. Indeed, Mrpl44GFP was found to interact with Mrpl44FLAG but not FLAGBak (Fig 2A). This suggests that Mrpl44 can exist in dimers or multimers.

HPLC was performed on whole cell extracts from wildtype NIH3T3 cells to determine if Mrpl44 only forms a dimer or is part of a larger complex. Endogenous Mrpl44 was found to elute as two different complexes, predominantly one of greater than 670kDa and another smaller one that eluted in the <158 kDa range (Fig 2B). Both correspond to complexes that are much larger than predicted Mrpl44 dimers. The elution of the larger Mrpl44 complex follows Mrpl12 and Mrps15, known components of the mitoribosome. However, it is also possible that Mrpl44 dimer/multimers may exist separately from the mitoribosome, either independently or in complex with subcomponents of the mitoribosomes [26].

To confirm that Mrpl44 is indeed part of the mitoribosome, Mrpl44FLAG and FLAGBak, as a negative control, were captured with αFLAG beads and the bound RNA was extracted. The presence of mitochondrial rRNA was then determined by quantitative RT-PCR. We found that both the 12S and 16S rRNAs were co-IPed with Mrpl44 (Fig 2C). In addition, Mrpl12, another component of the large subunit of the mitoribosome, co-IPed with Mrpl44, along with Mrps15, a component of the small subunit (Fig 2D). This confirms that Mrpl44 is indeed associated with the mitoribosome.

### Expression of mtDNA-encoded genes is compromised by Mrpl44 knockdown in NIH3T3 fibroblasts

**Fig 3:**
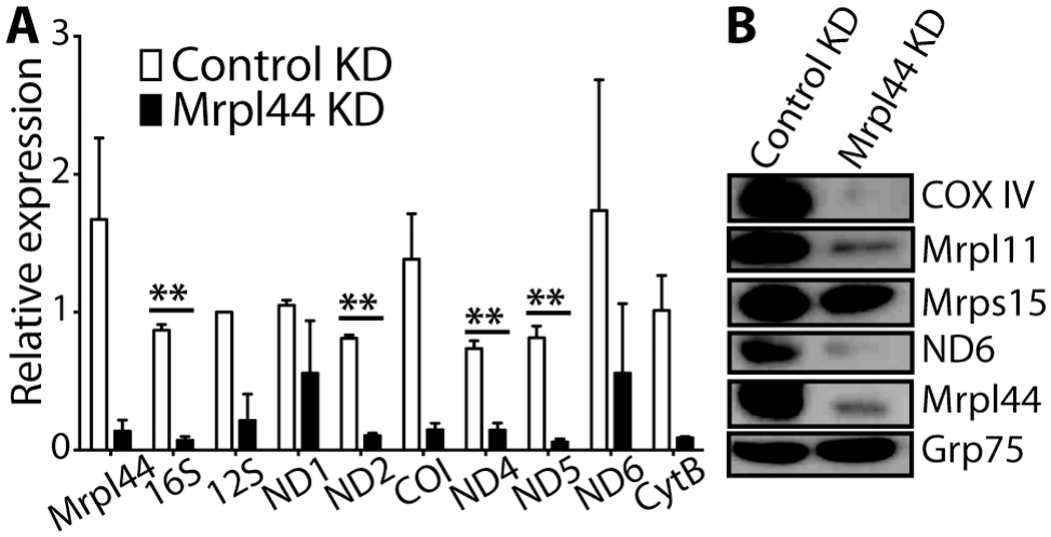
Knockdown of Mrpl44 affects the expression of mtDNA-encoded genes. Protein and RNA were extracted from NIH3T3 knocked down (ORF) for Mrpl44 or expressing a control shRNA. (A) RNA expression of mitochondrial genes was analyzed by quantitative RT-PCR. Expression was normalized to β-actin. Statistical analysis was performed using multiple t-test with Holm-Sidak correction for multiple comparisons (**p<0.005). The mean +/- SEM of 3 independent experiments is shown. (B) Western blotting for the OXPHOS proteins COX IV and ND6, mitoribosome proteins Mrpl44, Mrpl11 and Mrps15. The mitochondrial protein Grp75 was analysed as a loading control. A representative of three independent experiments is shown.

Given the localization of Mrpl44 to the matrix of the mitochondria and its association with the mitoribosome, we sought to determine if Mrpl44 is required for the expression of the mtDNA. We investigated the impact on mtDNA-encoded RNA and protein expression levels in NIH3T3 cells following perturbation of Mrpl44 levels. Knockdown of Mrpl44 by stably transduced shRNAs resulted in decreased levels of mtDNA-encoded RNA. 16S rRNA, ND2, ND4 and ND5 mRNA transcripts, in particular, were significantly decreased (Fig 3A). On the other hand, when Mrpl44 was overexpressed, mtDNA-encoded RNA was increased (S3 Fig). These results indicate that Mrpl44 affects the level of RNA within the mitochondria.

The downregulation in mitochondrial RNA levels corresponded to decreased protein levels of the mtDNA-encoded protein ND6, OXPHOS system Complex IV (COX IV), and another protein associated with the large subunit of the mitoribosome, Mrpl11 (Fig 3B). However, expression of the small subunit of the mitoribosome Mrps15 and the nDNA-encoded mitochondrial protein Grp75 were unaffected. Mrpl44 overexpression did not affect mitochondrial protein expression (S3 Fig). These results indicate that Mrpl44 plays a role in the regulation of mitochondrial-encoded protein expression.

### Mitochondrial genome copy number is unaffected by altered Mrpl44 expression

It is possible that impact on mitochondrial gene expression caused by altered Mrpl44 level may be via changes in mitochondrial genome copy number. To examine this, genomic DNA was extracted from NIH3T3 cells over-expressing Mrpl44GFP or knocked down for Mrpl44. qRT-PCR analysis for mitochondrial versus nuclear DNA found that altered Mrpl44 expression had no impact on the mitochondrial DNA content of cells (S4 Fig). Thus, Mrpl44 appears to regulate mitochondrial genome expression rather than copy number.

### Mrpl44 affects immature mitochondrial RNA transcript levels

We next investigated whether altered Mrpl44 levels might impact the maturation of mitochondrial genome-encoded transcripts by measuring expression of the immature mitochondrial RNA transcripts (i.e. polycistronic transcripts that are yet to be processed). This was down by qRT-PCR with using primers flanking the 5’ and 3’ ends of mitochondrial tRNAs. We found that knockdown of Mrpl44 resulted in the accumulation of the tRNA Glu junction (S5 Fig), while overexpression resulted in reduction in a range of tRNA junctions (S5 Fig). This suggests that the presence of Mrpl44 reduces the amount of immature mitochondrial RNA transcripts. This could potentially be by enhancing transcript maturation.

### Mitochondrial translation in NIH3T3 fibroblasts is impaired by Mrpl44 knockdown

**Fig 4:**
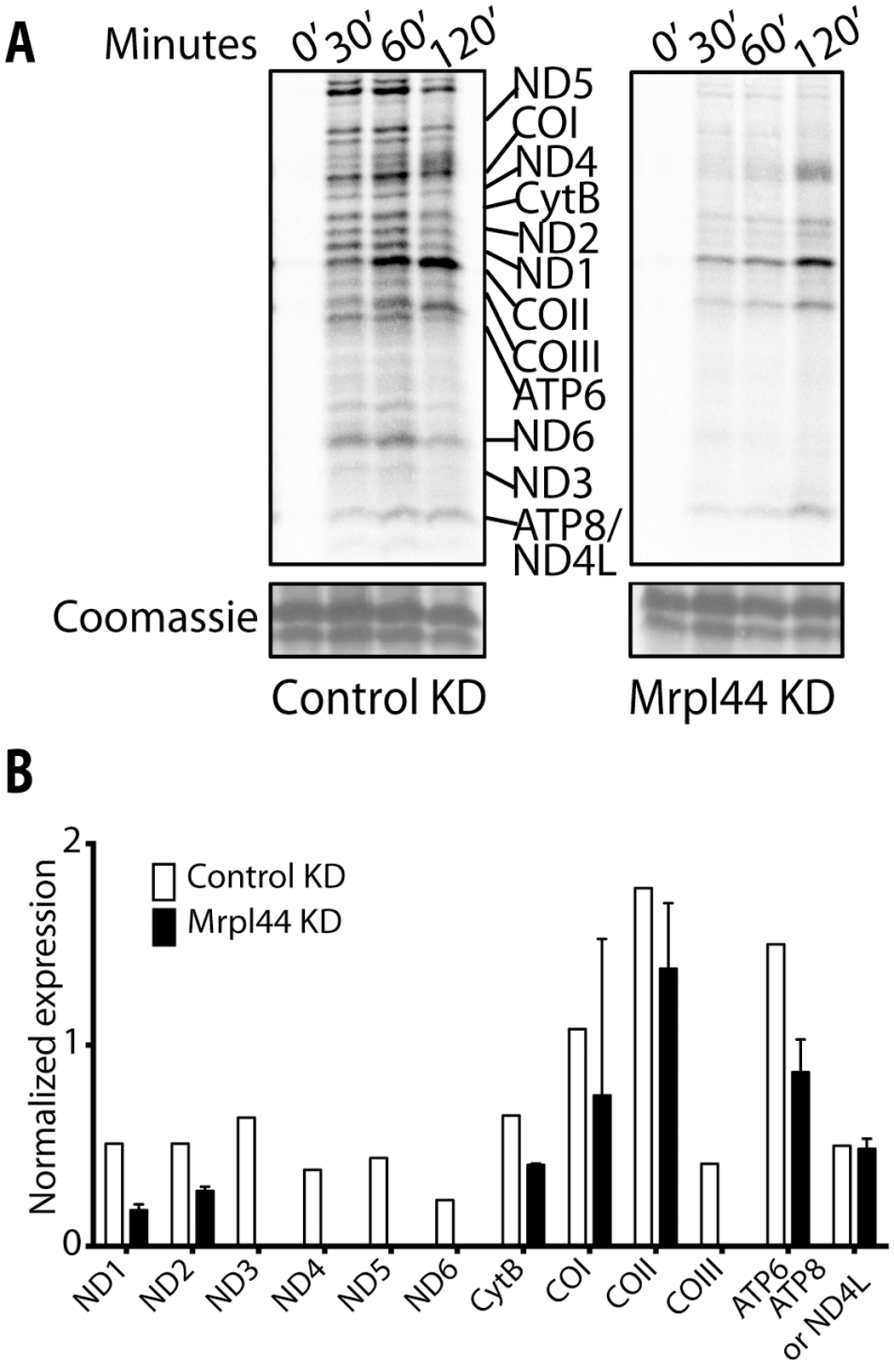
Knockdown of Mrpl44 decreases mitochondrial translation. (A) NIH3T3 cells knocked down for Mrpl44 (ORF) or expressing a control shRNA were radiolabelled with ^35^S-methionine for 120mins. Cytosolic translation was inhibited by the addition of emetine. Protein extracts were collected at 0, 30, 60 and 120 mins after ^35^S-methionine addition. The extracted mitochondrial proteins were separated on a 10–16% gradient SDS-PAGE then autoradiographed. To validate equal protein loading, a small section of the gel was stained with Coomassie Blue. One or two experiments is shown. (B) Densitometry analysis of the bands at 120 mins. The data were normalized to the control COII expression at 30 mins. The mean +/- SD of 2 different shRNA knockdowns (ORF and 3’UTR) is shown.

We next examine whether Mrpl44 might also regulate mitochondrial translation, rather than just affecting transcript maturation. A mitochondrial translation assay was performed. ^35^S-methionine was used to radiolabel mitochondrial translation products in the presence of emetine, which inhibits cytosolic translation. Knockdown of Mrpl44 resulted in a clear decrease in overall mitochondrial protein translation, with fewer proteins being labeled during the time course (Fig 4A). Further quantification revealed that the subunits ND3, ND4, ND5, ND6 and COIII were below detection limits with Mrpl44 knockdown (Fig 4B). Of the proteins detected during the time course, the rate of translation was decreased as well, with the subunits ND1, ND2, CytB and COI being labeled 30 min later, at the 60 min time point, compared to control cells (S6 Fig). These results indicate that Mrpl44 does not only affect the mtDNA at the RNA level, but also at the level of protein translation.

### Mrpl44 knockdown impairs Complex I- and Complex II- dependent ATP synthesis

The regulation of mitochondrial protein translation by Mrpl44 is likely to impact mitochondrial function. To determine if altered Mrpl44 expression affects ATP synthesis, we measured the maximal capacity of Complex I- and Complex II-dependent ATP synthesis in digitonin permeabilised NIH3T3 cells with altered Mrpl44 levels. These measurements were obtained under optimal substrate conditions: i) with the Complex I-dependent substrates glutamate and malate, in the presence or absence of the Complex I inhibitor rotenone; or ii) with the Complex II-dependent substrate succinate (with rotenone to force forward electron transfer from Complex II), in the presence or absence of the Complex II inhibitor malonate. Rates were then normalized to the control Complex II-dependent rate (succinate + rotenone) in each experiment.

**Fig 5:**
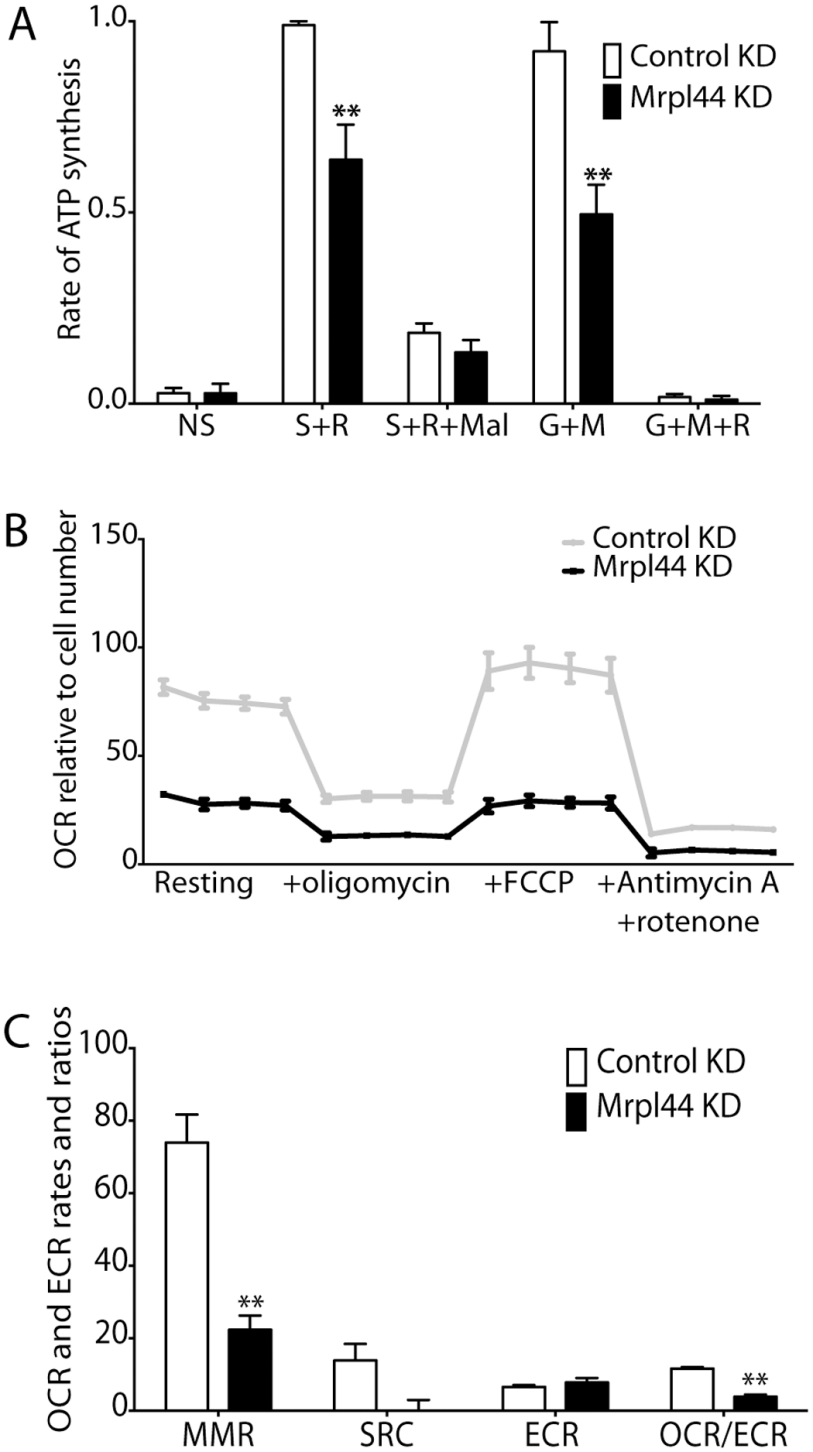
ATP synthesis capacity and bioenergetics are compromised by Mrpl44 knockdown. (A) Rates of ATP synthesis were measured in NIH3T3 cells knocked down (ORF) for Mrpl44 or expressing a control shRNA, in the presence of specific substrate inhibitor combinations. Rates are expressed relative to the control Complex II-dependent rate (succinate + rotenone) on the day. The mean +/- SEM from six independent experiments is shown. (B) Oxygen consumption rates (OCR) measured by Seahorse XF24/3 extracellular flux analysis. The values were normalized to cell number, determined by CyQUANT. Four measurements were taken at each stage of the assay. (C) Calculation of maximum respiratory rate (MRR), spare respiratory capacity (SRC), extracellular acidification rate (ECAR) and OCR/ECAR ratio from the Seahorse analysis. The mean +/- SEM of 3 independent experiments is shown. Statistical analysis performed using multiple t-test with Holm-Sidak correction for multiple comparisons (**p<0.005).

Knockdown of Mrpl44 resulted in a reduction in Complex I- and Complex II-dependent rates of ATP synthesis (Fig 5A). This correlates with the reduced assembly of the OXPHOS Complexes I, III, IV and V. For both Mrpl44 KD and control cells, the Complex I and Complex II-dependent ATP synthesis were abolished by the appropriate inhibitors (Fig 5A), confirming that measurement of ATP synthesis was “substrate-specific”. ATP synthesis rates were unchanged with Mrpl44 overexpression (S7 Fig).

### Mrpl44 expression level affects mitochondrial respiratory capacity

To better characterize the impact of altered Mrpl44 expression on OXPHOS, we analyzed the OCR of cells in culture. Consistent with impaired ATP synthesis, the resting OCR, maximal OCR (MRR), and spare respiratory capacity (SRC) were reduced with the KD of Mrpl44 (Fig 5B and 5C). Interestingly, although we could not detect an impact of Mrpl44 overexpression on ATP synthesis, OCR, MRR and SRC were increased (S7 Fig). We also monitored the ECAR as an indirect measure of glycolytic activity, but found no impact of altering Mrpl44 expression (Fig 5C and S7 Fig). This is consistent with Mrpl44 affecting the expression of the mtDNA-encoded components of the OXPHOS system. The OCR to ECAR ratio (OCR/ECAR) is an indication of the preference of the cell to utilize OXPHOS versus glycolysis to generate ATP. This ratio was reduced in Mrpl44 KD fibroblasts; the heavier reliance on glycolysis is not surprising as OXPHOS is severely compromised in these cells (Fig 5C). This again confirms that Mrpl44 is critical for expression of the mtDNA-encoded components of the OXPHOS system.

## Discussion

Mitochondrial disorders represent a large number of diseases and are generally caused by OXPHOS dysfunction [27]. As the OXPHOS subunits are encoded by both nuclear and mitochondrial genes, inheritance of OXPHOS diseases can either be due to sporadic mutations, autosomal recessive or dominant, X-linked or maternal [28]. In recent years, it has become clear that defects in mitochondrial gene transcription and translation underlie many of these syndromes [29]. Mitochondrial defects caused by mtDNA-encoded genes are generally easy to identify. However, the mitochondrial transcription and translation machinery is primarily encoded by the nuclear genome (apart from the rRNAs). And until recently, mapping the mutations in these nuclear-encoded mitochondrial genes has been difficult. With the advent of high throughput sequencing techniques, an increasing number of these mutations in nuclear mitochondrial genes have been identified [5,30–34]. For instance, autosomal recessive mutations in translation elongation factors cause phenotypes such as encephalopathy and cardiomyopathy (TSFM) and leukoencephalopathy (TUFM) [35–37].

A number of mutations that affect mitochondrial translation have been identified. This include mutations in the core MRPs, including MRPS16 [38], MPS22 [39]and MRPL12 [40], as well as in several accessory proteins, such as ObgH1 [41] and C7orf30 [42]. Mutations in MRPS16 and MRPS22 appear to affect the stability of the mitoribosome [38,39], while MRPL12 appears to be involved in the recruitment of elongation factors as well as the modulation of mitochondrial gene expression [40] [43]. Impaired translation can affect the assembly of all or only specific complexes within the OXPHOS pathway [38–42]indicating that mitochondrial genome translational is a highly regulated process. In the case of MRPL44, it is clear that altered expression levels affect overall mitoribosome levels. However at present, it is unclear if it at the level of assembly or stability of the large mitoribosome subunit. The decrease in the 16S rRNA suggests perhaps a defect in the assembly of the large subunit. This is correlates with previous findings [24]. A defective large subunit of the mitoribosome is thus consistent with the impairment of translational observed when the loss of Mrpl44.

As putative RNase III protein, whether Mrpl44 has a role in RNA processing remains unclear. The mitochondrial infantile cardiomyopathy caused by MRPL44 mutation in humans has been localized to the p.L156R residue within the RNase III domain [24]. Comparative analysis of MRPL44 to the solved RNase III structure of *A*. *aeolicus* [24,44] suggests that L156R is not involved in RNA binding, but potentially important for maintaining protein structure [24]. It was predicted that the mutation causes incorrect folding of MRPL44 and thus reduced expression. This, unfortunately, does not inform us of the function of the RNase III of Mrpl44. However, it clearly indicates the importance of this protein for mitochondrial function. It is quite possible that other mutations that impart partial loss of function, rather than just affect expression levels, may contribute to other mitochondrial syndromes.

Given the structural similarities between Mrpl44 and RNase III in bacteria, we hypothesized that it might exist in dimeric form. Our data indicates that Mrpl44 may indeed form dimers or multimers. The key question is whether Mrpl44 has a role in the processing of dsRNAs like all other RNase III proteins. The associations with mitochondrial RNAs that we observed are consistent with a potential function in RNA processing. A possible role of Mrpl44 could be in the regulation or maturation of mtDNA-encoded RNA products. While bioinformatics analysis showed that the critical residues for RNase activity are missing in mammalian Mrpl44 [8,24], whether this is truly enzymatically dead remains to be determined. More detailed characterization of the Mrpl44 interacting factors or the analysis of mitochondrial-wide RNA processing products in Mrpl44 mutant cells may be illuminating. Regardless, given the implications on mitochondrial infantile cardiomyopathy, further investigations into the function of Mrpl44 are clearly warranted.

## Supporting Information

S1 Fig Mrpl44 contains a simple structure similar to bacterial RNase III. (A) The RNase III—dsRBM (RNC module) of murine Drosha was blasted on ExPASy [45] to search for potentially novel mammalian RNase III proteins. Shown is the alignment of the amino acid sequence of the RNC module of murine Drosha, Mrpl44 and E. coli RNase III. Conserved residues are highlighted in red. (B) The simple structure of Mrpl44 appears to be more similar to bacterial RNase III than to Drosha or Dicer. Shown is a schematic representation of the domains found in RNase III enzymes.
(PDF)

S2 Fig Immunofluorescence microscopy of control GFP cells. Shown are NIH3T3 fibroblasts, CommaD P24 mammary epithelial cells and N2A neuronal cells expressing free GFP. The mitochondria were co-stained with MitoTrackerRed and the nuclei with DAPI. The cells were analyzed by wide-field microscopy (400X magnification).
(PDF)

S3 Fig Overexpression of Mrpl44 levels affects the expression of mitochondrial- encoded genes. Protein and RNA was extracted from NIH3T3 cells that are over-expressing Mrpl44GFP or GFP only. (A) RNA expression of mitochondrial genes was analyzed by quantitative RT-PCR. Expression was normalized to β-actin. Statistical analysis was performed using multiple t-test with Holm-Sidak correction for multiple comparisons (**p<0.005). The mean +/- SEM of three independent experiments is shown. (B) Western blotting for mitochondrial proteins. A representative of three independent experiments is shown.
(PDF)

S4 Fig Alteration of Mrpl44 level does not affect mitochondrial genome copy number. Genomic DNA was extracted from NIH3T3 over-expressing Mrpl44GFP or knocked down for Mrpl44 (ORF). Nuclear versus mitochondrial DNA levels were analyzed by quantitative RT-PCR. The mean ± SEM of two independent experiments is shown. Statistical analysis was performed using multiple t-tests with Holm-Sidak correction for multiple comparisons. Primers amplifying fragments within the *Drosha* and *Mir17* loci were used to quantify the nuclear genome, while primers amplifying fragments with 16S and CytB were used to quantify the mitochondrial genome.
(PDF)

S5 Fig Altered Mrpl44 levels affect immature mitochondrial RNA transcript levels. RNA was extracted from (A) NIH3T3 knocked down for Mrpl44 (ORF) or expressing a control shRNA and (B) NIH3T3 cells that are over-expressing Mrpl44GFP or GFP only. Detection of immature transcripts was performed by quantitative RT-PCR measurement of the tRNA junctions between genes. The mean ± SEM of three independent experiments is shown. Statistical analysis performed using multiple t-tests with Holm-Sidak correction for multiple comparisons (*p<0.05).
(PDF)

S6 Fig Knockdown of Mrpl44 decreases rate of mitochondrial translation. NIH3T3 cells knocked down for Mrpl44 or expressing a control shRNA were radiolabelled with ^35^S- methionine for 120mins. Protein extracts were collected at 0, 30, 60 and 120 mins after ^35^S- methionine addition. The extracted mitochondrial proteins were separated by 10–16% gradient SDS-PAGE and autoradiographed. Shown is the densitometry analysis of the bands from Fig 4. The data were normalized to the control COII expression at 30 mins. The mean ± SD of two different Mrpl44 shRNAs (ORF and 3’UTR) is shown.
(PDF)

S7 Fig ATP synthesis capacity and bioenergetics of NIH3T3 fibroblasts with Mrpl44 overexpression. (A) Rates of ATP synthesis were measured in NIH3T3 cells over-expressing Mrpl44GFP or GFP only, in the presence of indicated substrate inhibitor combinations. Rates are expressed relative to control CII-dependent rate (S+R) on the day. The mean +/- SEM from six independent experiments is shown. (B) Oxygen consumption rates (OCR) in NIH3T3 cells overexpressing Mrpl44GFP or GFP only were measured by Seahorse XF24/3 extracellular flux analysis. The values were normalized to cell number, by CyQUANT. Four measurements were taken at each stage of the assay. (C) Calculation of maximum respiratory rate (MRR), spare respiratory capacity (SRC), extracellular acidification rate (ECAR) and OCR/ECAR ratio from the Seahorse analysis. The mean +/- SEM of three independent experiments is shown. Statistical analysis performed using multiple t-test with Holm-Sidak correction for multiple comparisons (*p<0.05; **p<0.005).
(PDF)

S1 Table Antibodies. Listed are details of the antibodies for the Western blotting analyses performed.
(PDF)

S2 Table Primer pairs. Listed are the primer pairs used for quantitative PCR analysis performed. Also PCRs were quantified by Sybr Green detection.
(PDF)
