# Supplementary material for: A Role for the Mitochondrial Protein Mrpl44 in Maintaining OXPHOS Capacity
Source: PLoS One. 2015 Jul 29;10(7):e0134326. doi: 10.1371/journal.pone.0134326 (PMC4519308; doi:10.1371/journal.pone.0134326)

A Alignment of the RNC domains (RNAseIII + DSRM)—conserved only

```
RNC       1
Mrpl44    1 DLLKTAFINSCYIKSEEAKRQSLGIEKEAALLNLKDNQELFEQGLSFSH
Drosha    1 RLLARAFT....LRTVGFNHLTLGHNQRMEFL...........GDSIMQ

RNC       1 YVIANALYHRFPRVDEGDMSRMRATLVRGNTLAELAREFELGECLRLGP
Mrpl44   50 RCLTQFLEDEFPDLPAEGTESLVSFLTGEAVVCHVARNLAVEQLTLSAE
Drosha   35 LVATEYLFIHFPDHHEGHLTLLRSSLVNNRTQAKVAEELGMQEYAITND

RNC      50 GELKSGGFRRESILADTVEALIGGVFLDSDIQTVEKLILNWYQTRLDEI
Mrpl44   99 FPVPLPVLR......QTFFAVIGALLQSSGPERAALFIRDFLITQMTGK
Drosha   84 KTKRPVALR.TKTLADLLESFIAALYIDKDLEYVHTFMNVCFFPRLKEF

RNC      99 SPGDKQ..KDPKTRLQEY.....LQGRHLPLPTYLVVQVRGEAHDQEFT
Mrpl44  142 ELFEMWTVVNPMGLLVEE.....LKKRNISAPESRLTRQSGS....TTA
Drosha  132 ILNQDW..NDPKSQLQQCCLTLRTEGKEPDIPLYKTLQTVGPSHARTYT

RNC     141 IHCQVSG..........LSEPVVGTGSSRR
Mrpl44  182 LPLYFVG..........LYCDRKLIAEGPG
Drosha  179 VAVYFKGERIGCGKGPSIQQAEMGAAMDALEK
```

Conserved in 3
Conserved in 2

B

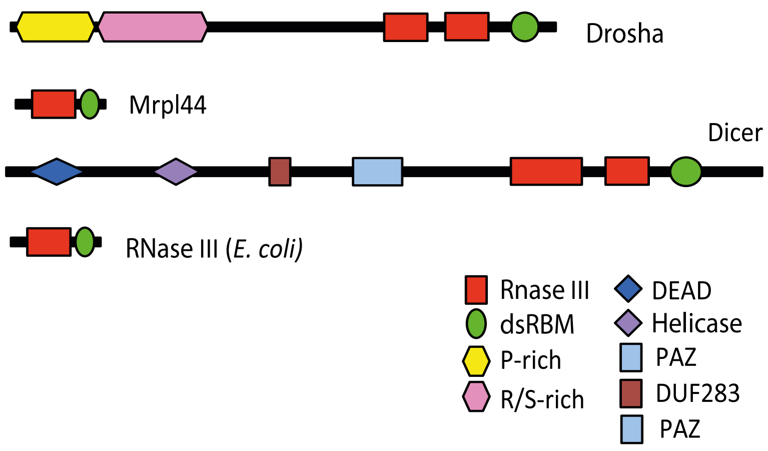

Supplement: S1 Fig — (A) The RNase III—dsRBM (RNC module) of murine Drosha was blasted on ExPASy [45] to search for potentially novel mammalian RNase III proteins. Shown is the alignment of the amino acid sequence of the RNC module of murine Drosha, Mrpl44 and E. coli RNase III. Conserved residues are highlighted in red. (B) The simple structure of Mrpl44 appears to be more similar to bacterial RNase III than to Drosha or Dicer. Shown is a schematic representation of the domains found in RNase III enzymes. (PDF) [file pone.0134326.s001.pdf]
