# Supplementary figures and images for: A Role for the Mitochondrial Protein Mrpl44 in Maintaining OXPHOS Capacity
Source: PLoS One. 2015 Jul 29;10(7):e0134326. doi: 10.1371/journal.pone.0134326 (PMC4519308; doi:10.1371/journal.pone.0134326)

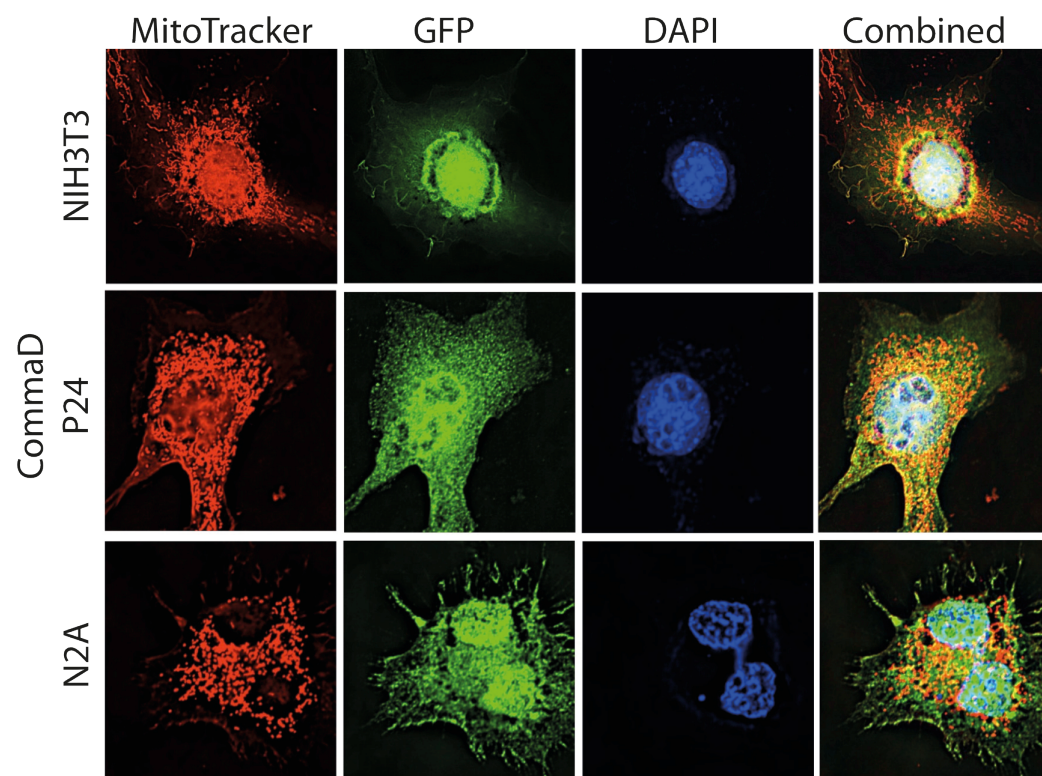

Supplement: S2 Fig — Shown are NIH3T3 fibroblasts, CommaD P24 mammary epithelial cells and N2A neuronal cells expressing free GFP. The mitochondria were co-stained with MitoTrackerRed and the nuclei with DAPI. The cells were analyzed by wide-field microscopy (400X magnification). (PDF) [file pone.0134326.s002.pdf]

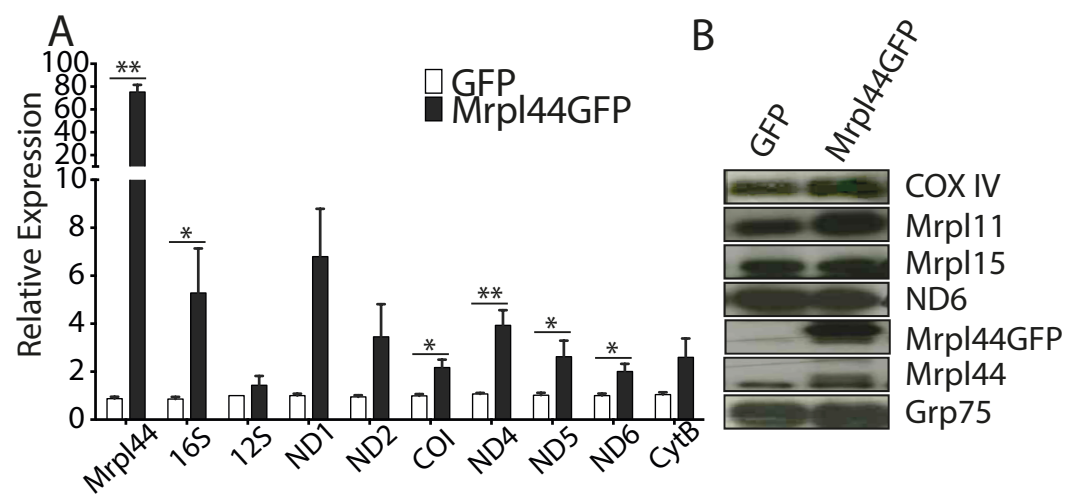

Supplement: S3 Fig — Protein and RNA was extracted from NIH3T3 cells that are over-expressing Mrpl44GFP or GFP only. (A) RNA expression of mitochondrial genes was analyzed by quantitative RT-PCR. Expression was normalized to β-actin. Statistical analysis was performed using multiple t-test with Holm-Sidak correction for multiple comparisons (**p<0.005). The mean +/- SEM of three independent experiments is shown. (B) Western blotting for mitochondrial proteins. A representative of three independent experiments is shown. (PDF) [file pone.0134326.s003.pdf]

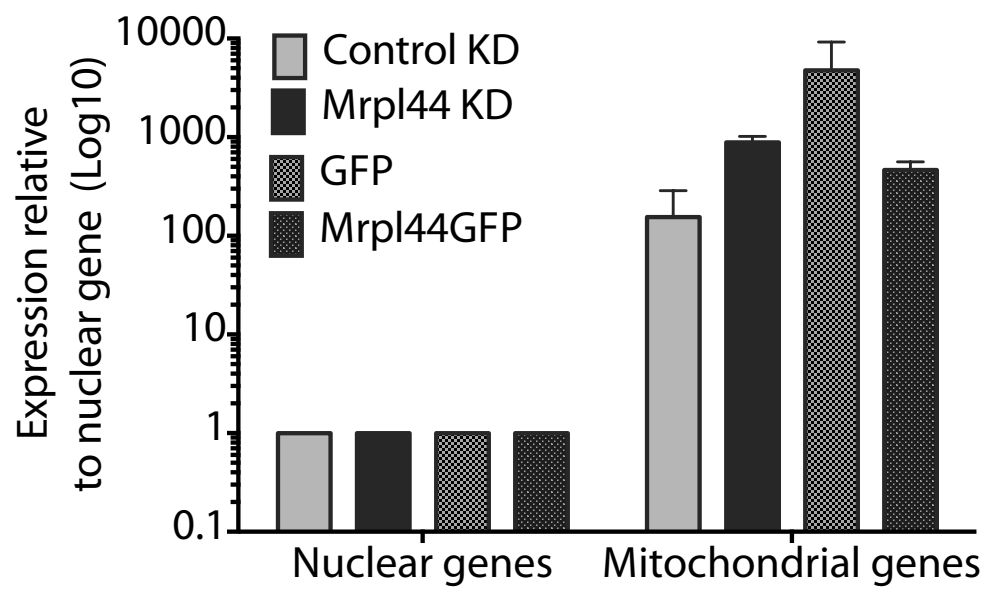

Supplement: S4 Fig — Genomic DNA was extracted from NIH3T3 over-expressing Mrpl44GFP or knocked down for Mrpl44 (ORF). Nuclear versus mitochondrial DNA levels were analyzed by quantitative RT-PCR. The mean ± SEM of two independent experiments is shown. Statistical analysis was performed using multiple t-tests with Holm-Sidak correction for multiple comparisons. Primers amplifying fragments within the Drosha and Mir17 loci were used to quantify the nuclear genome, while primers amplifying fragments with 16S and CytB were used to quantify the mitochondrial genome. (PDF) [file pone.0134326.s004.pdf]

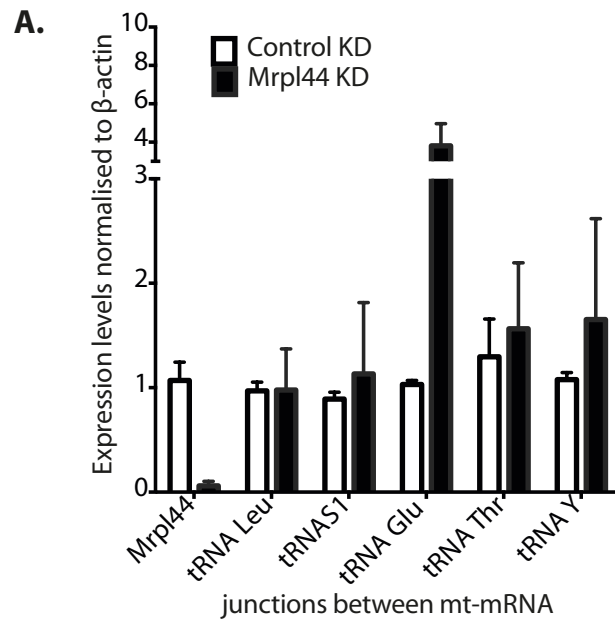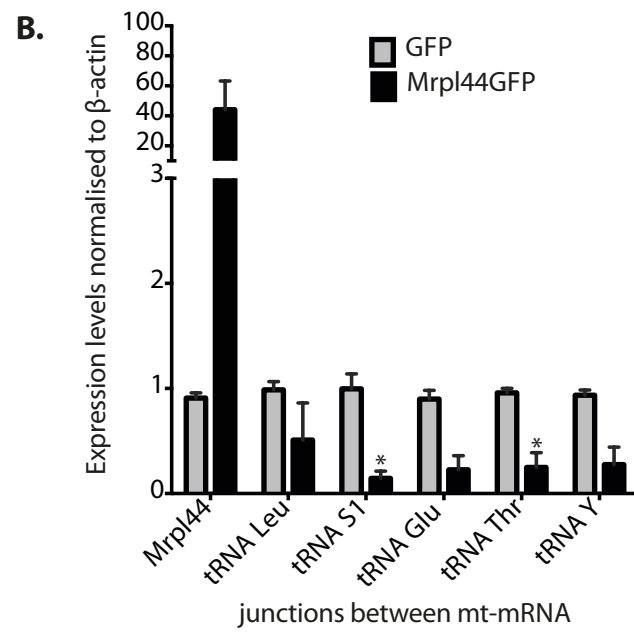

Supplement: S5 Fig — RNA was extracted from (A) NIH3T3 knocked down for Mrpl44 (ORF) or expressing a control shRNA and (B) NIH3T3 cells that are over-expressing Mrpl44GFP or GFP only. Detection of immature transcripts was performed by quantitative RT-PCR measurement of the tRNA junctions between genes. The mean ± SEM of three independent experiments is shown. Statistical analysis performed using multiple t-tests with Holm-Sidak correction for multiple comparisons (*p<0.05). (PDF) [file pone.0134326.s005.pdf]

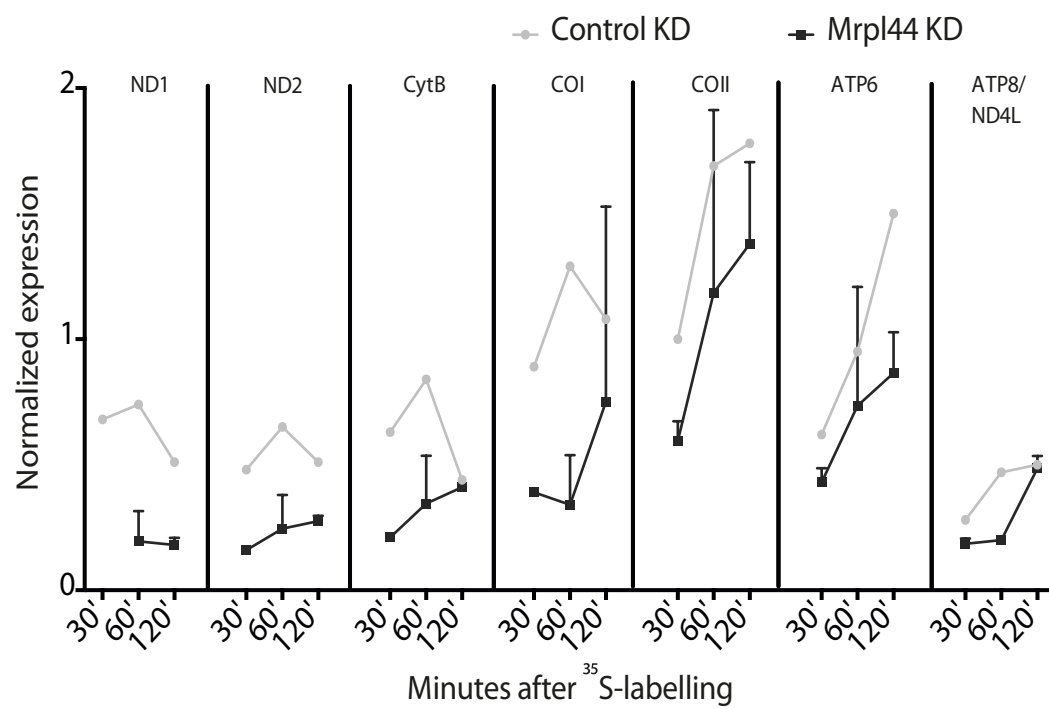

Supplement: S6 Fig — NIH3T3 cells knocked down for Mrpl44 or expressing a control shRNA were radiolabelled with 35S- methionine for 120mins. Protein extracts were collected at 0, 30, 60 and 120 mins after 35S- methionine addition. The extracted mitochondrial proteins were separated by 10–16% gradient SDS-PAGE and autoradiographed. Shown is the densitometry analysis of the bands from Fig 4. The data were normalized to the control COII expression at 30 mins. The mean ± SD of two different Mrpl44 shRNAs (ORF and 3’UTR) is shown. (PDF) [file pone.0134326.s006.pdf]

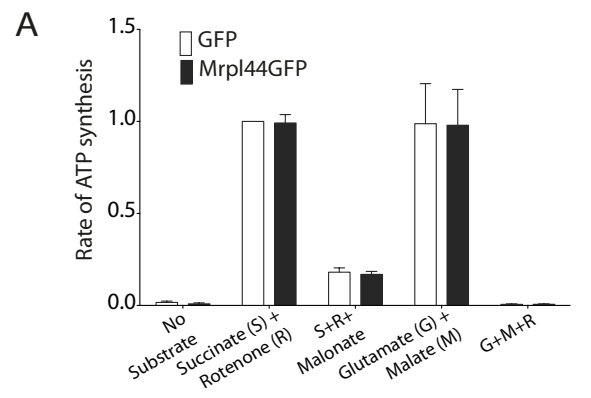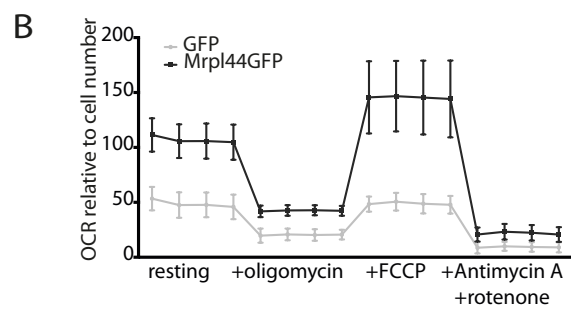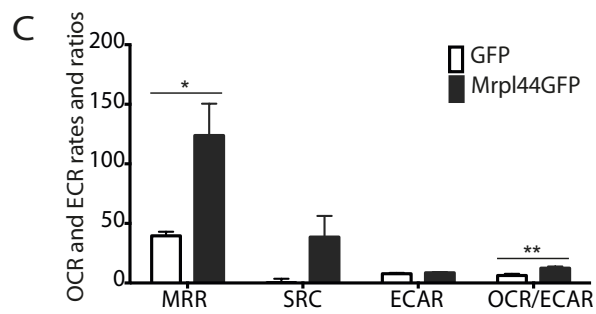

Supplement: S7 Fig — (A) Rates of ATP synthesis were measured in NIH3T3 cells over-expressing Mrpl44GFP or GFP only, in the presence of indicated substrate inhibitor combinations. Rates are expressed relative to control CII-dependent rate (S+R) on the day. The mean +/- SEM from six independent experiments is shown. (B) Oxygen consumption rates (OCR) in NIH3T3 cells overexpressing Mrpl44GFP or GFP only were measured by Seahorse XF24/3 extracellular flux analysis. The values were normalized to cell number, by CyQUANT. Four measurements were taken at each stage of the assay. (C) Calculation of maximum respiratory rate (MRR), spare respiratory capacity (SRC), extracellular acidification rate (ECAR) and OCR/ECAR ratio from the Seahorse analysis. The mean +/- SEM of three independent experiments is shown. Statistical analysis performed using multiple t-test with Holm-Sidak correction for multiple comparisons (*p<0.05; **p<0.005). (PDF) [file pone.0134326.s007.pdf]
