# Supplementary material for: A Role for the Mitochondrial Protein Mrpl44 in Maintaining OXPHOS Capacity
Source: PLoS One. 2015 Jul 29;10(7):e0134326. doi: 10.1371/journal.pone.0134326 (PMC4519308; doi:10.1371/journal.pone.0134326)
Supplement: S1 Table — Listed are details of the antibodies for the Western blotting analyses performed. (PDF) [file pone.0134326.s008.pdf]

**Antibody**	**Clone**	**Concentration used**	**Company**
Flag	M2	1:1000	Sigma
Mrpl44	16394-1-AP	1:1500	Proteintech
Mrpl11	15543-1-AP	1:600	Proteintech
Mrpl12	14795-1-AP	1:500	Proteintech
Mrps15	17006-1-AP	1:1500	Proteintech
MT-ND6	sc-20510-R	1:600	Santa Cruz
GFP	#2956	1:1000	Cell Signaling
Grp75	30A5	1:5000	Enzo Life Science
