# Supplementary material for: A Role for the Mitochondrial Protein Mrpl44 in Maintaining OXPHOS Capacity
Source: PLoS One. 2015 Jul 29;10(7):e0134326. doi: 10.1371/journal.pone.0134326 (PMC4519308; doi:10.1371/journal.pone.0134326)
Supplement: S2 Table — Listed are the primer pairs used for quantitative PCR analysis performed. Also PCRs were quantified by Sybr Green detection. (PDF) [file pone.0134326.s009.pdf]

Target	Forward	Reverse
COI	5’-CTGAGCGGGAATAGTGGGTA	5’-TGGGGCTCCGATTATTAGTG
ND1	5’-GGATCCGAGCATCTTATCCA	5’-GGTGGTACTCCCGCTGTAAA
ND2	5’-AGGGATCCCACTGCACATAG	5’-CTCCTCATGCCCCTATGAAA
ND4	5’-CAATCTGCTTACGCCAAACA	5’-GCTGTGGATCCGTTCGTAGT
ND5	5’-ATAACCGCATCGGAGACATC	5’-GAGGCCAAATTGTGCTGATT
ND6	5’-GGGTTTGGTGGATCGTTTTT	5’-ACCAATCTCCCAAACCATCA
Cytb	5’-ATTCCTTCATGTCGGACGAG	5’-ACTGAGAAGCCCCCTCAAAT
16S	5’-GGGATAACAGCGCAATCCTA	5’-GATTGCTCCGGTCTGAACTC
12S	5’-ACCGCGGTCATACGATTAAC	5’-CCCAGTTTGGGTCTTAGCTG
tRNA Leu	5’-CAATCCAGGTCGGTTTCTATCTA	5’-GTCGACAACAATGTTAGGGCCTTTTCG
tRNA S1	5’- AGAGGCCTTTGCTTCAAAAC	5’- GTCGACAGACCAAGTTGGAATGGGTA
tRNA Glu	5’- AGGTGAAGGCTTTAATGCTAAC	5’- GTCGACGACCTGTAATGATTTGGACTATTAGG
tRNA Thr	5’- CCAACCAGTAGAACACCCAT	5’- GTCGACAGTTTAATTAGAATACCAGCTTTGGG
tRNA Y	5’- GCTAAATACCCTATTACTGGCTT	5’- GTCGACAAAAGCATGGGCAGTTACGATA
